# 7-Tesla MRI demonstrates absence of structural lesions in patients with vestibular paroxysmia

**DOI:** 10.3389/fnana.2015.00081

**Published:** 2015-06-09

**Authors:** Paulus S. Rommer, Gerald Wiest, Claudia Kronnerwetter, Heidemarie Zach, Benjamin Loader, Kirsten Elwischger, Siegfried Trattnig

**Affiliations:** ^1^Department of Neurology, Medical University of ViennaVienna, Austria; ^2^Department of Biomedical Imaging and Image-guided Therapy, Centre of Excellence, High-Field MR, Medical University of ViennaVienna, Austria; ^3^Department of Otorhinolaryngology, Head and Neck Surgery, Rudolfstiftung Tertiary Teaching HospitalVienna, Austria; ^4^Rehabilitation Center MeidlingVienna, Austria

**Keywords:** high field MRI, vestibular paroxysmia, neuroscience, nerve compression, neuro imaging, clinical neurology, anatomy

## Abstract

Vestibular parxoysmia (VP) is a rare vestibular disorder. A neurovascular cross-compression (NVCC) between the vestibulochochlear nerve and an artery seems to be responsible for short attacks of vertigo in this entity. An NVCC can be seen in up to every fourth subject. The significance of these findings is not clear, as not all subjects suffer from symptoms. The aim of the present study was to assess possible structural lesions of the vestibulocochlear nerve by means of high field magnetic resonance imaging (MRI), and whether high field MRI may help to differentiate symptomatic from asymptomatic subjects. 7 Tesla MRI was performed in six patients with VP and confirmed NVCC seen on 1.5 and 3.0 MRI. No structural abnormalities were detected in any of the patients in 7 Tesla MRI. These findings imply that high field MRI does not help to differentiate between symptomatic and asymptomatic NVCC and that the symptoms of VP are not caused by structural nerve lesions. This supports the hypothesis that the nystagmus associated with VP has to be conceived pathophysiologically as an excitatory vestibular phenomenon, being not related to vestibular hypofunction. 7 Tesla MRI outperforms conventional MRI in image resolution and may be useful in vestibular disorders.

## Introduction

Vestibular paroxysmia (VP) is a recently defined vestibular syndrome (Brandt and Dieterich, 1994). Affected patients usually suffer from short attacks of vertigo lasting from seconds to few minutes. A neurovascular cross-compression (NVCC) is assumed to be responsible for the symptoms.

In patients presenting with typical symptoms a contact between the vestibulochochlear nerve and an artery (mostly the anterior inferior cerebellar artery [AICA]) can be visualized in almost all patients on magnetic resonance imaging (MRI; Yurtseven et al., 2004; Gultekin et al., 2008). Over time caloric testing may disclose a mild unilateral vestibular asymmetry. Furthermore, hyperventilation-induced nystagmus was found in a majority of patients (Hüfner et al., 2008; Brandt et al., 2010). Studies have shown that decompression of the NVCC led to clinical improvement of the vertigo (Møller et al., 1993). Strupp et al presented a patient with VP and right peripheral vestibular deficit caused by a compression of the nerve (Strupp et al., 2013). A structural lesion of the vestibulocochlear nerve seems to be possible but has not been shown so far. The relevance of an NVCC is not completely clear, as it can be seen in up to about every fourth subject in magnetic resonance imaging (MRI), but not all subjects may suffer from typical symptoms (Gultekin et al., 2008). Thus, structural alterations as a sign of nerve lesions should be made visible.

The use of MRI in detecting nerve compression and structural lesions has been explored recently (Gambarota et al., 2007; Leal et al., 2010; Thawait et al., 2012). Advances in MRI techniques imply that 7 Tesla (T) MRI may be an appropriate tool to detect neural lesions caused by nerve compression due to its higher spatial resolution and higher contrast agent sensitivity. Signal sensitivity on T1 weighted MR sequences is increased in 7 T MRI due to longer T1 relaxivities, compared to 3 T MRI. Its high spatial resolution and enhanced contrast sensitivity has led to new pathophysiological insights in diseases like multiple sclerosis, brain tumors and vascular diseases (Noebauer-Huhmann et al., 2010; van der Kolk et al., 2011, 2012, 2013).

The purpose of the current study was to assess possible structural lesions of the vestibulocochlear nerve in patients with VP by means of 7 T MRI and to find out whether 7 T MRI may help to differentiate between symptomatic and asymptomatic NVCC.

However, 7 T MRI has some limitations. First, new techniques and image protocols have to be implemented to visualize the regions of interest. Second, anatomical structures vary between individuals and third, visualization depends on the compliance of the subjects and the skills of the technician in the planning and execution of sequences as well as the postprocessing skills. Fourth, and most important, nerve lesions are the consequences of the pressure of the vessel in individual patients. Pressure will vary between patients and structural alterations may be present at different levels which may not be distinguishable by 7 T MRI.

## Material and Methods

The study was approved by the local ethical committee (221/2010) and local review board. All patients gave their informed and written consent to participate in the study. Imaging protocols were established based on experiences in a healthy volunteer who gave written and informed consent. After the implementation of the imaging protocol patients with VP were enrolled in the study.

The diagnosis of VP has been established according to the Hüfner criteria (Table 1). 7 T MRI of the brain was performed in all patients with VP. Inclusion criteria required a confirmed NVCC in 1.5 T or 3 T MRI.

**Table 1:** **Diagnostic criteria as suggested by Hüfner et al. (2008)**.

At least five episodes of recurrent vertigo fulfilling all of the following criteria:
A) Episodes of vertigo last from seconds to a few minutes
B) Episodes occur:
at rest
at certain body/head positions (no BPPV-maneuvres)
changes with body/head positions (no BPPV-maneuvres
C) One of the following characteristics at attacks
disturbance of gait
disturbance of stance
no accompanying symptoms
unilateral tinnitus
unilateral pressure or numbness in or around an ear
unilateral reduced hearing
D) One or several additional diagnostic criteria:
NVCC demonstrated on MRI (CISS sequence)
hyperventilation-induced nystagmus
increase of vestibular deficit as measured on follow-up investigations by ENG
Treatment response to antiepileptics
E) No other causal explanation

Six consecutive patients from the Neurootology Outpatient Clinic, Department of Neurology, Medical University Vienna, diagnosed as having VP were enrolled in this study. All patients reported recurrent short attacks of vertigo lasting a few seconds up to a few minutes. Patients were symptomatic for several months up to three decades. All patients had an NVCC as shown by 1.5 or 3 T MRI CISS sequence and all patients reported improvement of symptoms after the administration of antiepileptic drugs. All patients underwent neurotological assessment. None of the patients showed spontaneous nystagmus. One patient showed abnormalities on head impulse test, suggesting a lesion on the left side (patient 2 from Table 2).

**Table 2:** **Patients with vestibular paroxysmia who underwent 7-Tesla-MRI**.

	Sex	Age	Therapy	Response to drug therapy	NVCC
1	Female	49	Gabapentin	Yes	Both sides
2	Female	46	Gabapentin	Yes	Left side
3	Male	47	Gabapentin,	No	Right side
			Oxcarbazepine	Insufficient
			Carbamezepine	Yes
4	Male	29	Gabapentin	Yes	Right side
5	Male	54	Gabapentin	Yes	Right side
6	Female	39	Gabapentin	No	Both sides
			Carbamazepine	Yes

### MRI Measurement

MR examination was performed on a 7 T whole body Siemens Magnetom MR scanner (Siemens Healthcare, Erlangen, Germany) with a maximal gradient strength of 40 mT/m and a slew rate of 200 T/m/s using a 8 canal Rapid head coil (RAPID Biomedical GmbH, Rimpar, Germany). T1-sequences (3 dimensional [D] MPRAGE = Magnetization Prepared Rapid Gradient Echo) were performed without and with intravenously administered MR contrast agent (gadolinium [multihance®]).

#### Imaging Protocol

The following sequences were applied:

Isotropic 3D MPRAGE sequence in the axial plane with the parameter as follows: TE/TR = 3.5/4000 ms, IR (inversion recovery) = 1700 (Non-sel.IR), 448 × 431 × 274 matrix size, FOV = 230 × 173 × 128 mm^3^, yield an isotropic 0.5 mm resolution one concatenation GRAPPA (generalized autocalibrating partially parallel acquisition) factor 2, in an acquisition time of 12:46.

The 2D-FSE T2 sequence in the axial plane with the parameter as follows: TE/TR = 81/4500 ms, Flip angle = 144°, 25 slices with 40% Distance factor, 688 × 688 matrix size, FOV = 230 × 173 mm, Voxel size = 0.3 × 0.3 × 1.5 mm, one concatenation, GRAPPA factor 2.4 averages, in an acquisition time of 8:48.

#### Image Postprocessing

MPR Images (multi planar reformat) were reconstructed from the isotropic MPRAGE series with 1.5 mm slices thickness.

## Results

**Figure 1:**
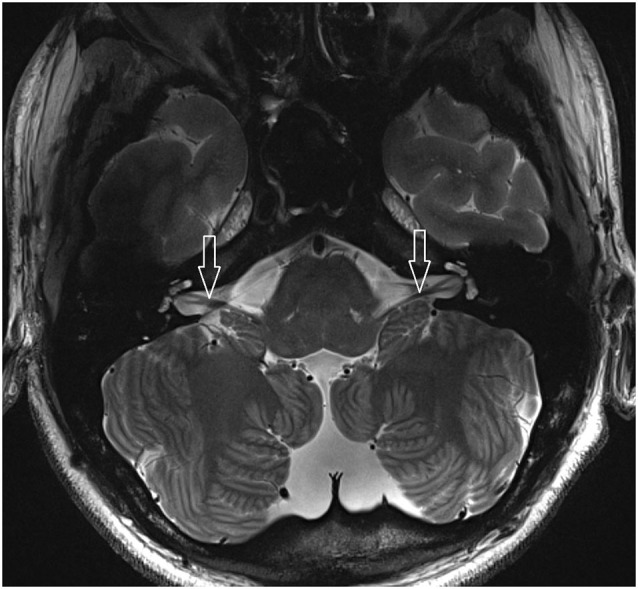
**2D FSE T2 axial with 1.5 mm slices thickness (healthy volunteer)**. White arrow: vestibulocochlear nerve.

**Figure 2:**
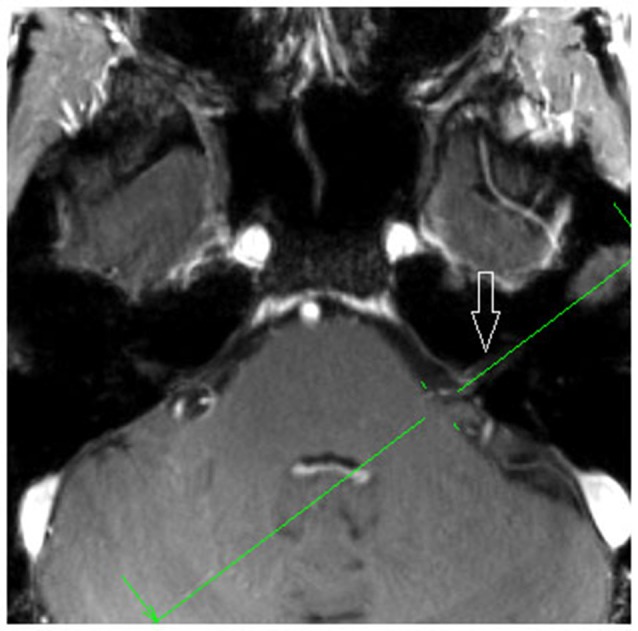
**Axial MPR with 1.5 mm slice thickness, showing the planning orientation from Figure 2b (green line)**. White arrow: vestibulochochlear nerve (patient 6).

**Figure 3:**
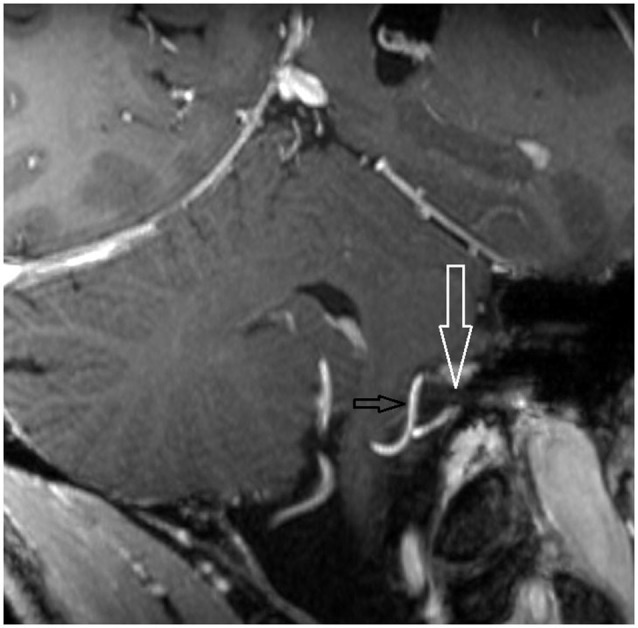
**Oblique coronal MPR with 1.5 mm slice thickness**. White arrow: vestibulochochlear nerve. Black arrow: AICA. (patient 6 left side).

**Figure 4:**
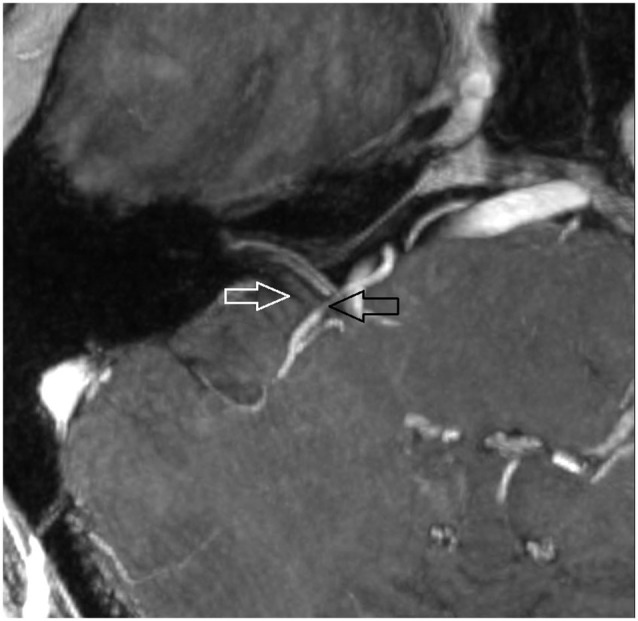
**Oblique coronal MPR with 1.5 mm slice thickness**. White arrow: vestibulochochlear nerve. Black arrow: AICA. (patient 6 right side).

**Figure 5:**
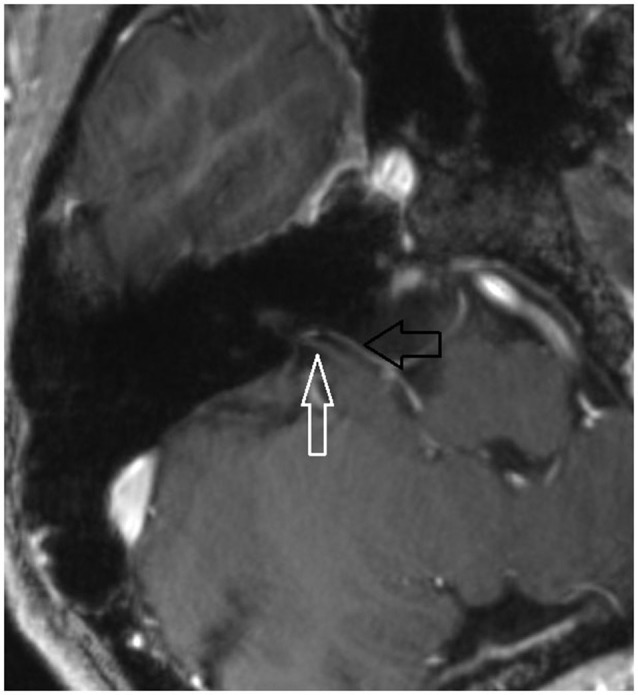
**Axial MPR with 1.5 mm slice thickness**. White arrow: vestibulochochlear nerve. Black arrow: AICA. (patient 3 right side).

7 T MRI was able to visualize the vestibulocochlear nerve in all subjects. Figure 1 shows the vestibularcochlear nerve in the healthy volunteer. Additionally, the division of the vestibular and cochlear parts of the nerve could be best visualized on T2 sequences. Reconstruction of MPRAGES images (MPR) allowed better visualization of the NVCC (see Figure 2). Reconstruction was based on the course of the vestibulocochlear nerve. This allowed focused visualization of the nerve and the contact between the nerve and the vessel. An NVCC between the AICA and vestibulocochlear nerve could be shown in all patients. NVCC was found on the left side in one patient, on the right side in three patients (see Figure 5) and in two patients on both sides (see Figures 3, 4). In none of the patients neither hyperintensities, nor contrast enhancement could be detected in the vestibulocochlear nerve on T2-FSE sequence or T1-weighted high resolution imaging. However, the high sensivitiy allowed for visualization of detailed anatomical structures (see Figure 1) and the relation between the nerve and vessels (Figures 2–5) was made visible.

## Discussion

As a novelty, this study is the first that utilized high field MRI in the visualiziation of brain structures involved in vestibular control. Additionally, we applied the sequences derived from a healthy volunteer to investigate the vestibularcochlear nerve on a series of patients with VP. By means of 7 T MRI it was possible to visualize the vestibularcochlear nerve in high resolution. Additionally, in all patients an NVCC between the vestibulocochlear nerve and the AICA could be detected, thus confirming the results from the previously performed 1.5 T and 3.0 T MRI investigations. Interestingly, in none of our patients structural lesions or contrast-enhancement of the symptomatic vestibulocochlear nerve could be detected. An anatomical contact of a vessel with the vestibulocochlear nerve has been shown in up to 25% of individuals undergoing MRI in previous studies, but not all suffered from VP-typical symptoms (Gultekin et al., 2008; van der Kolk et al., 2012) All of our patients, however, fulfilled the Hüfner criteria (Hüfner et al., 2008) and reported VP-typical symptoms. Furthermore, all our patients responded to the administration of antiepileptic medication.

There are some possible explanations as to why no structural lesions could be seen in our study.

One might hypothesize that MRI is only partially suitable in detecting structural nerve lesions. However, MRI investigations have shown to be effective in the detection of nerve compressions and associated structural abnormalities. 7 T MRI is a highly sensitive method to detect structural lesions in the central and peripheral nervous system (Noebauer-Huhmann et al., 2010). By MRI it is possible to distinguish between intra- and perineural masses and thus to visualize discontinuities in peripheral nerves. Hyperintensities on T2 images results from prolonged T2 relaxation time and are signs for structural pathologies (Filler et al., 1996; Leal et al., 2010). Gadolinium-enhancement has been shown to be a pathognomonic feature of median nerve compression at an early stage (Du et al., 2010) and thus seems to be an appropriate method to detect abnormalities of peripheral nerves at an early stage. In patients with trigeminal neuralgia, NVCC may lead to microstructural changes (lower fractional anisotropy and higher apparent diffusion coefficient) due to vascular compression. These structural changes may induce nerve atrophy (Leal et al., 2011; Lutz et al., 2011; Chen et al., 2012). Nerve pathologies might be a combination of focal or diffuse enlargement of the nerve in MRI. Over the last decades MRI has gained importance in the evaluation and diagnostics of peripheral nerve pathology (Kobayashi et al., 2009). However, besides the NVCC no further pathologies could be detected in our patients.

Thus, we propose another explanation for the absence of structural lesions. Structural lesions are not a binary entity with a simple “yes” or “no” result. Structural lesions results from a complex interplay of the nerves and the vessels. The effects of external nerve compression and the associated nerve pathology depend strongly on the amount of the induced pressure. Irreversible structural nerve lesions can be induced at pressure levels of 80 mg Hg. Pressures of about 20 mg Hg will impair the venous supply of nerves, while pressures of 40 mg Hg will reduce the arterial supply (Subhawong et al., 2012). The associated congestion and edema can be visualized as hyperintensity on T2 images. A long lasting compression finally results in inflammation and fibrosis, which is best shown on postcontrast T1 images (Subhawong et al., 2012). Depending on the individual compression, abnormalities may be detected in patients with VP by 7 T MRI.

Our findings imply that the symptoms of VP in our patients are obviously not caused by an NVCC with associated high pressure, as none of our patients showed structural abnormalities of the vestibulocochlear nerve on 7 T MRI. It rather seems that a neurovascular contact (and possible mild structural abnormalities that are not detectable on 7 T MRI) are sufficient to induce VP-typical symptoms. These findings are also of pathophysiological importance, as the associated nystagmus during VP can thus be understood as an excitatory nystagmus (induced by abnormal irritation of the vestibulocochlear nerve) and not as a lesion-induced nystagmus, as seen in unilateral vestibular loss (e.g., vestibular neuritis). The absence of evident structural nerve lesions in VP also explains why—even after decades of symptoms—antiepileptic drugs can cause complete remission of VP.

Our study also confirms the feasibility of 7 T MRI examination in patients with vestibular dysfunction. Vertigo is known to occur in healthy subjects undergoing MRI examinations by influencing the labyrinthine endolymphatic fluid (Roberts et al., 2011). None of our patients reported severe dizziness during or after MRI, demonstrating that even ultra-high-field MRI is well tolerated in patients with VP.

In conclusion, 7 T MRI confirmed the pathologic NVCC as shown by CISS sequence in 1.5 T and 3 T MRI. In addition, the high anatomical resolution provided by 7 T allowed to exclude evident structural lesions of the vestibulocochlear nerve in all patients in this study. The results of our study suggest that 7 T MRI may not be able to differentiate between symptomatic and asymptomatic compressions of the vestibulocochlear nerve. Larger trials are needed to confirm our findings.

## Author Contributorship

Conception and design of the work: PSR, GW, BL, CK, ST. Acquisition, analysis, or interpretation of data for the work: PSR, GW, HZ, CK. Drafting the work or revising it critically for important intellectual content: PSR, GW, CK, BL, HZ, ST. Final approval of the version to be published: PSR, GW, CK, BL, HZ, ST. The following authors agree to be accountable for all aspects of the work in ensuring that questions related to the accuracy or integrity of any part of the work are appropriately investigated and resolved: PSR, GW, CK, BL, HZ, ST.

## Conflict of Interest Statement

The authors declare that the research was conducted in the absence of any commercial or financial relationships that could be construed as a potential conflict of interest.
